# Captive Breeding Programs Based on Family Groups in Polyploid Sturgeons

**DOI:** 10.1371/journal.pone.0110951

**Published:** 2014-10-30

**Authors:** Elisa Boscari, Jose Martin Pujolar, Isabelle Dupanloup, Riccardo Corradin, Leonardo Congiu

**Affiliations:** 1 Department of Biology, University of Padova, Padova, Italy; 2 Department of Bioscience, Aarhus University, Aarhus C, Denmark; 3 Institute of Ecology and Evolution, University of Berne, Berne, Switzerland; 4 Swiss Institute of Bioinformatics, Lausanne, Switzerland; 5 Department of Statistics, University of Padova, Padova, Italy; Nanjing Agricultural University, China

## Abstract

In species with long life cycles and discontinuous availability of individuals to reproduction, implementing a long-term captive breeding program can be difficult or impossible. In such cases, managing diversity among familiar groups instead of individuals could become a suitable approach to avoid inbreeding and increase the possibility to accomplish a breeding scheme. This is the case of several sturgeon species including the Adriatic sturgeon, whose recovery depends on the management of a few captive stocks directly descended from the same group of wild parents. In the present study, relatedness among 445 potential breeders was inferred with a novel software for pedigree reconstruction in tetraploids (“*BreedingSturgeons*”). This information was used to plan a breeding scheme considering familiar groups as breeding units and identifying mating priorities. A two-step strategy is proposed: a short-term breeding program, relying on the 13 remaining F0 individuals of certain wild origin; and a long-term plan based on F1 families. Simulations to evaluate the loss of alleles in the F2 generation under different pairing strategies and assess the number of individuals to breed, costs and logistical aquaculture constraints were performed. The strategy proposed is transferable to the several other tetraploid sturgeon species on the brink of extinction.

## Introduction

Conservation management aimed at preserving the genetic variability of wild populations is essential in preventing the extinction of endangered species [1], [2]. Heritable genetic variation is a prerequisite for the long-term survival of species since the rate of evolutionary change able to occur in a group of organisms depends on the amount of genetic variation present in the gene pool; hence, organisms exhibiting low levels of genetic variability will show an impaired evolutionary potential to face future environmental changes [3]. Given the imminent risk of extinction of most critically endangered species and the low number of reproductive populations available in nature, *in situ* conservation efforts (e.g. translocation, breeding in a protected area of wild habitat, supplementary feeding) are often not sufficient to reduce or prevent the effects of anthropogenic and stochastic threats [1]. Therefore, conservation activities must frequently rely on *ex situ* conservation through breeding of captive individuals [1], [3], [4], [5], [6].

Captive breeding has long played an important role in the conservation of threatened and endangered species and generally involves two basic principles. The first one is the complete genetic characterization of all breeders available in captivity in order to quantify the amount of genetic variation and to assess the purity of the stocks. Typically, captive stocks contain only a fraction of the original genetic variability present in the larger wild population. A second goal aims at retaining and transmitting most of the residual genetic diversity to the future generations of captive breeders, thus securing the long-term future of the species [7], [8].

In this regard, pedigree-based management practices are used to identify candidate breeders, *i.e.* the “*breeders unit*”, for the production of the future generations [9], [10]. Evidences from empirical studies suggest that the best strategy to retain gene diversity is to minimize average kinship when pairing individuals [11]. Therefore, an accurate pedigree of the captive breeders is critical, together with information on the relatedness among the original founder population, although in most captive programs founders are assumed to be equally unrelated (e.g. “founder assumption”) [5]. Due to their high variability, microsatellites are the most adequate marker of choice for parentage assignment in animal conservation [2], [5], [12].

Among fishes, the need of establishing long-term captive programs is especially urgent for sturgeons, which according to the recent report (March 2010) of the *International Union for Conservation of Nature* (IUCN) are the most threatened group of species in the world, with 85% of sturgeons being at risk of extinction (http://www.iucnredlist.org). The decline of sturgeon populations is mostly attributed to human activities, including overexploitation for the harvesting of caviar, habitat degradation, damming of rivers and poaching [13], [14]. So far, recovery actions have been based on supplementation of natural populations with juveniles produced in captivity from wild breeders. However, for several species/populations the low availability of wild animals makes the retention of part of the fingerlings produced necessary to be used as future breeders [15]. Some particular life-characteristics of sturgeons (*i.e.* slow growth, late age at maturity, 2-years or more resting stage between reproductions) complicate breeding plans as selected individuals might not be available when needed for reproduction. Moreover, sturgeons show high juveniles mortality, which might translate into different number of retained fingerlings from each reproduction that reaches maturity. This can result in broodstocks composed by different familiar groups with unbalanced sizes. Consequently, in the absence of pedigree information, the risk of mating close relatives can be high, thus increasing relatedness and distorting founder diversity [8].

With the aim of proposing adequate approaches for the management of broodstocks composed by familiar groups that differ in size and genetic composition, we focused on the Adriatic sturgeon (*Acipenser naccarii*), an endemism of the Adriatic region. Given its tetraploid status [16], we also aim at developing adequate tools for the management of tetraploid genetic data in pedigree-based breeding programs.

The Adriatic sturgeon has been listed by IUCN since May 2010 as “critically endangered” and possibly extinct in the wild (IUCN 2012). A breeding program was initiated in 1977 with the transfer of ca. 50 immature wild individuals (F0) to the private aquaculture plant Azienda Agricola V.I.P. (Orzinuovi, Brescia, Italy). Since the late 1980s, following the first successful reproduction in captivity [17], several F1 broodstocks have been established from randomly paired F0 breeders. Over 250,000 juveniles have been reintroduced in the last 25 years, and although recaptures have been occasional, up to now there is no evidence of natural reproduction. Therefore, the F0 stock at the V.I.P. aquaculture plant, currently reduced to 13 individuals, represents the only living animals of unequivocal wild origin. All Adriatic sturgeons reared in Europe directly descend from this limited stock. The recovery of the Adriatic sturgeon depends mostly on the management of F1 captive stocks that have now reached sexual maturity.

In the present study, we used a pedigree-based approach to conduct the characterization of the stocks available at the Azienda Agricola V.I.P. Plant, including (1) all remaining individuals of the parental F0 stock, and (2) a large number of F1 individuals retained from many breedings performed in the facilities during the last 30 years. After pedigree reconstruction using a new allocation program specific for tetraploid species (“*BreedingSturgeons*”), candidate breeders were identified. A breeding plan was then designed based on family groups as breeding units. The rationale of using families rather than individuals is based on the fact that selected individuals might not be available for reproduction when needed due to the particular complex life history traits of sturgeons (i.e. they reproduce every other year), but related individuals might be available instead. Our breeding program was also supported by simulations in order to estimate the loss of genetic diversity under different scenarios as well as to evaluate the sustainability of the plan from an economic and logistic point of view. The present approach can also be applied to many other species of sturgeons whose recovery depends on the establishment of *ex situ* strategies based on captive broodstock.

## Materials and Methods

### Sample collection and DNA extraction

The University of Padova ethic board C.E.A.S.A. (Comitato Etico di Ateneo per la Sperimentazione Animale) exempted this study from review because it was an extra moenia activity. Nevertheless, we tried to minimize the impact of sample collection on the animals. All samples were collected in aquaculture facilities by the owners. Fish were kept into the pond water to minimize stress and were released immediately after sample collection, which consisted of painless clippings from the caudal fin. No mortality, pain or stress was observed.

We analyzed a total of 42 individuals from the parental stock F0, already genotyped at 24 microsatellite loci [10]. All analyses were conducted considering the full data set (referred as Wild; N = 42) and considering only those individuals alive at present (referred as Wild-Alive, N = 13).

We also analyzed 445 F1 individuals. In July 2003, about half of the F1 individuals died due to a poisoning incident and only 233 animals survived. All analyses were conducted considering the full F1 data set (F1, N = 445) and considering those F1 alive at present (F1-Alive, N = 233).

Our analysis also includes 133 individuals from the Ticino River Park in Italy for comparison. This individuals are part of a stock established from a very limited number of breeders [18].

Genomic DNA was extracted from fin-clip (10–100 mg) using the DNA Easy Tissue Extraction Mini Kit (Euroclone) and stored at −4°C.

### Mitochondrial analysis

Prior to the analysis, species status of all individuals was assessed using mitochondrial DNA haplotypes plus species-specific SNPs at the S7 and Vimentin genes [19]. Amplification of the mitochondrial control region (d-Loop) was performed for all individuals using the primer pair PRO1F-PHE1R, following the standard experimental conditions [10]. All PCR products were purified by enzymatic reaction with ExoSAP-it (Usb) and sequenced at the external service BMR Genomics using an ABI Prism3730XL automatic sequencer. A multi-alignment was created using ClustalW in Mega5 [20] and a haplotype network was constructed using TCS v. 1.13 [21] following the statistical parsimony approach [22]. Genetic diversity was measured using haplotype diversity (H) and nucleotide diversity (Pi) estimated from number of segregating sites and from mean number of pairwise differences using DnaSP software v. 5 [23].

Finally, in order to detect the presence of other species or interspecific hybrids, the analyses of the mitochondrial control region was supplemented with the scoring of species-specific SNPs in two genes, the ribosomal Protein S7 and Vimentin, following the approach developed in [19].

### Microsatellite analysis

All F1 individuals were genotyped at 7 microsatellite loci: LS-39 [24], AnacE4, AnacC11, AnacA6 [25], An20 [26], AoxD234 and AoxD64 [27], selected among the 24 loci that were originally used to genotype the F0 stock [10]. In case of multi-allocations (when more than one parent pair resulted compatible), 3 additional microsatellite loci were genotyped, An16 [26], AoxD241 and AoxD161 [27]. Microsatellite loci were amplified following the conditions reported in the original references. Microsatellite scoring was performed using GeneMapper software version 1.95 (Soft Genetics LLS^R^). The interpretation of microsatellite patterns in tetraploids is not straightforward due to the presence of multiple alleles at each locus that can be present in more than one copy, thus the true genotype cannot be accurately resolved. As an alternative, we used the classic band-sharing approach as proposed in [10] which considers presence/absence of bands, disregarding the number of alleles present in each individual. Data from all microsatellite loci were combined by creating individual profiles where bands were coded as a string of 0 s (absent) and 1 s (present). Band sharing (BS) between individuals was used as a measure of similarity. Pairwise genetic distances were calculated as 1-BS. Number of alleles at the different loci was used to compare F1 and F0 stocks and to evaluate the efficiency of allele transmission.

### Pedigree reconstruction

A new software (“*BreedingSturgeons*”) was implemented to estimate an allocation compatibility index for each F1 individual to all possible parent pairs in the F0 stock, assuming a tetrasomic mode of inheritance with no dominance [28].

For all possible pairs of putative males and females in the F0 stock and the F1 individuals, we inferred all possible tetraploid genotypes compatible with the observed microsatellite profiles at all loci. We defined a single locus compatibility index, *ci*(x), of an individual to a putative parent pair as: *ci*(x) = ∑[A(p1)+A(p2)]/4, where *A(pi)* is the number of alleles that could have been inherited from parent-*i*. *A(pi)* can vary between 0 (if no allele present in parent i is seen in the possible tetraploid genotypes inferred for the F1 individual) and 2 (if 2 alleles could have been transmitted from that parent to the F1 individual). And ci(x) can vary between 0 and 1. Multi locus estimates of compatibility indexes were obtained as average across all microsatellites loci. In this procedure the following assumptions must be satisfied: a) all the offspring alleles should be shared with the parental pair, b) two of the offspring alleles must be shared with the mother and two with the father, c) a complete heterozygote can not transmit two copies of the same allele. The above conditions should be satisfied by at least one of the inferred genotypes in the two parents and in the offspring.

Each F1 individual was allocated to the parent pairs associated with a compatibility index above a threshold (0,857) chosen to account for possible genotyping errors at one locus.

Individuals that could not be assigned to any parent pairs (not-allocated) probably represent the progeny of wild F0 individuals died before the characterization of the F0 stock. All individuals allocated with a compatibility index higher than the threshold value were checked for mitochondrial concordance with the putative mother.

We also used the program to test the power of the set of 7 microsatellite loci used for parental allocation. To do so, we generated 1,000 virtual F1 profiles from the F0 individuals. We identified the allocation power of our microsatellite panel as the fraction of virtual F1 profiles unequivocally allocated back to the correct parental pair.

In order to identify closely related individuals (full-sibs or half-sibs) among not-allocated animals, distributions of pairwise genetic distances between full- and half-sibs were created and threshold values under which 99% of comparisons between animals with a given degree of relatedness were identified [10]. The threshold values estimated for our panel of loci (0.58 for sibs and 0.66 for half-sibs) were applied to the distance matrix between F1 individuals to identify putative groups of related individuals.

### Breeding strategy

A “whole-family” approach (rather than a single-breeder approach) was used. The parent pair of each family was considered to be representative of the genetic diversity of the corresponding progeny. This allowed using a higher amount of genetic information since all the F0 individuals are genotyped at 24 loci [10]. Accordingly, the genetic distance between two families is represented by the genetic distance between the two cumulative parental profiles.

In the case of families with unknown pedigree, estimation of genetic distances from the cumulative profile of parents was not possible. In these cases, parental profiles were inferred cumulating the phenotypes of their offspring using the 7 loci genotyped for parental allocation analysis. Genetic distances estimated on these partial profiles were included in the matrix after observing a significant positive correlation (r = 0,770; p<0.001) between distances estimated at 24 and 7 loci by using the Mantel test implemented in PopTools [29].

The strategy proposed for the breeding plan is based on the following three criteria: (a) prioritize families with higher genetic value, (b) exclude crosses between families with a shared parent and (c) avoid already-performed crosses. Priority of the different families (named “*priority families*”) for reproduction was estimated based on how often the parents of each family were already represented in the F1 “*breeders unit*”. This value was estimated by averaging the number of progeny of the two parents across all the families in the “*breeders unit*”. In this way, the smallest families with under-represented parents were the first to be selected for reproduction. The above values were then converted into an ordinal scale (“priority”). Following the established order, each priority family was then paired with the more distant one (named “*mating family*”) selected from among all of the families available in the “breeders unit”.

With the aim of excluding crosses between half-sibs families, starting from the F0 profiles at 24 loci, all the possible combinations of virtual families (cumulative profiles) sharing one parent were created and the pairwise genetic distances estimated. The highest distance observed (0.35) was used as threshold value to exclude all crosses involving a shared parent. In the selection of the mating families we imposed the constraint that each *mating family* could not be crossed with more than 3 *priority families*. Consequently, the maximum number of crosses in which a given family can be involved is 4 (3 as “*mating family*” and 1 as “*priority family”*). This choice was necessary to avoid that some families with a high genetic distance from many others were used in too many crosses. This process was implemented in the R software “*BreedingPlanSturgeons*” also used to compare the different pairing strategies described in the following paragraph.

For the families whose parents were unknown, the information at 7 loci was used, which yielded a threshold value of 0.45. These families were excluded from the automated analyses and for each of them the more distant “*mating family*” was manually identified and a single cross suggested.

### Simulations

A first R-script, “*CostsBreedingSturgeons*” (script available as additional material), was developed to infer the number of crosses that can be simultaneously performed in a given hatchery. It includes the estimate of costs and yields of a given breeding program based on the number of individuals generated and reared until release. It also considers that at the beginning of the third year (after tagging of all individuals), a fraction of the individuals are retained in the facilities in order to preserve the captive stock, while the rest of individuals can be released into the wild.

We took into consideration the following constraints: number of individuals that can be induced every year, availability of ponds for rearing families separately until tagging and maximum number of animals that can be hosted. We also considered biological features (*e.g.* mortalities and growth rates at different life stages) and costs (*e.g.* reproductions, food, tagging, manipulation, overheads) in order to estimate the total financial cost of the project. Additional variables are reported in Table A in File S1. Logistic factors can be modified according to the capacities of the different hatcheries and the biological features of the species under consideration. In our example, the different variables were based on the “Storioni Ticino hatchery” located in Cassolnovo (Pavia, Italy), where the F1 V.I.P. Stock will be maintained in the future. The run was performed assuming six tanks available for the first two years of rearing and a common pond of 1,200 square meters for the following years. Biological parameters such as average female fecundity (30,000 eggs), hatching rate (50%) and mortalities rates in the first (87%), second (66%) and following years (10% every year) were based on personal communication by the V.I.P. owners, who have more than 20 years of experience in the reproduction of Adriatic sturgeon in aquaculture.

A second R-script, “*BreedingPlanSturgeons*” (also available as additional material), was developed to estimate the expected fraction of alleles of the F1 stock that are successfully transmitted to the progeny under different breeding strategies. To this purpose, the program “*BreedingSturgeons*” (previously described and used for parental allocation) was applied to generate a virtual F2 progeny from the observed F1 microsatellite phenotypes. The minimum number of individuals to be crossed in order to ensure the transmission of all F1 alleles was estimated. Based on the order of the “*priority families*”, four different scenarios were simulated in which the “*mating families*” were chosen by “Maximum Distance”, selecting 1, 2, 3 or 4 individuals per family (variables “nInd.Prior” and “nInd.inc” in Table B in File S1). For each scenario, 100 replicates (variable “nRep” in Table B in File S1) were performed and the mean cumulative number of alleles successfully transmitted was counted.

Once the optimal number of individuals per family was fixed, we also tested three alternative strategies to select and pair families: random selection of both paired families (Random – Random) vs. priority order of the first family with “*mating family*” selected at random (Priority – Random) or with “*mating family*” selected for having the higher genetic distance (Priority – Maximum distance). The process of random selection of the “*mating family*” was repeated 100 times for each “*priority family*” and for each resulting combination breeders were randomly extracted from the two families. Also the extraction of the breeders was repeated 100 times for each family to minimize possible effects of differences due to intra-family diversity. Finally, from each virtual cross, 20 fingerlings (variable “nSons” in Table B in File S1) were generated and the total number of alleles counted. The total number of alleles successfully transmitted during the process was averaged among replicates and used to compare the different pairing strategies. The choice of generating 20 virtual individuals is consistent with the number of animals that will be retained in captivity as future breeders from each reproduction.

In all the above simulations, the sex of animals of the “*breeders unit*” was not considered since this information was not available. Moreover, all families for which less than 24 loci were genotyped were excluded from the simulations. For the above simulations RStudio was used.

## Results

### Genetic diversity

Prior to the analysis, species status of all individuals was assessed using mitochondrial DNA haplotypes (Table 1) plus species-specific SNPs at the S7 and Vimentin genes. In total, two hybrids were identified (one hybrid white sturgeon female × Adriatic sturgeon male and one hybrid Adriatic sturgeon female × white sturgeon male) and were consequently removed from the analysis.

**Table 1:** Mitochondrial diversity indices.

Sample	N	S	S_i_	H	H_d_	θ_w_	θ_π_
**Wild**	43*	13	3	7	0.783	0.004	0.006
**Wild-Alive**	13	9	0	4	0.744	0.004	0.006
**F1**	443	10	0	5	0.665	0.002	0.002
**F1-Alive**	230	10	0	4	0.681	0.002	0.002

Diversity indices for all samples including number of individuals (N), number of segregating sites (S), number of singletons (S_i_), number of haplotypes (H), haplotype diversity (H_d_), nucleotide diversity estimated from number of segregating sites (**θ**_w_) and nucleotide diversity estimated from mean number of pairwise differences (**θ**_π_). Samples include the original parental F0 stock (Wild), the current F0 stock (Wild-Alive), the full F1 data set excluding the two detected hybrids (F1) and those F1 alive at present (F1-Alive). *Includes one heteroplasmic female that was considered as two females with different haplotypes.

The number of individuals showing each haplotype in the two stocks (F1/F1-alive) were: 0/0; 118/53, 220/106, 0/0, 75/45, 4/0, 37/23 for haplotype 1 to 7 respectively. A total of 11 individuals (one female F0 and 10 of its progeny) were heteroplasmic, presenting a double peak in the chromatogram sequence, and were counted as two different haplotypes for the analysis. When comparing genetic diversity across generations (Table 1), the F1 sample showed a decrease in haplotype diversity (F1: H = 0.67; F1-Alive: H = 0.68) compared to the parental stock of wild origin (Wild: H = 0.78; Wild-Alive: H = 0.74). Two out of the 7 original F0 haplotypes [19] were lost (hapl-1 and hapl-4) due to the random selection of mating pairs, while a third haplotype was lost after the 2013 poisoning event. Similarly, nucleotide diversity declined significantly when comparing the F0 and F1 samples, with dropping from 0.006 to 0.002.

Measures of genetic diversity at microsatellite loci are detailed in Table 2. Number of alleles ranged from 8 to 14 across loci in the wild population. A drop in the average number of alleles was observed when comparing the original Wild and the Wild-Alive (F0) stock, from 10.29 to 8.29. However, the F1 stock showed similar allele numbers in comparison with the original F0 stock (10 before poisoning; 9.86 after poisoning). The lowest average number of alleles (8.57) was observed in the F1-Ticino stock reared at the Ticino River Park. It is worth noting that some of the alleles observed in the F1 stocks were not observed in the F0 generation.

**Table 2:** Number of alleles per locus in the different stocks and average band sharing (BS) values at 7 microsatellite loci.

Locus	Wild(N = 42)	Wild–Alive(N = 13)	F1(N = 443)	F1-Alive(N = 231)	F1-Ticino(N = 133)
**LS-39**	8	8	8/1	8/0	7/0
**AoxD64**	9	8	9/1	9/1	9/0
**AnacE4**	10	7	10/3	10/3	8/0
**AnacC11**	11	7	9/1	9/1	8/0
**AoxD234**	14	12	14/0	14/0	13/0
**An20**	11	9	11/1	11/0	8/2
**AnacA6**	9	7	9/0	8/0	7/0
**Mean**	10.29	8.29	10/1	9.86/0.71	8.57/0.29
**BS**	0.40	0.39	0.45	0.45	0.50

Samples include the original parental F0 stock (Wild), the current F0 stock (Wild-Alive), the full F1 V.I.P. data set (F1) and those F1 alive at present (F1-Alive). For the F1 stocks, the values presented correspond to alleles shared with the F0 generation and newly observed alleles, respectively. Detected hybrids were excluded from the analysis.

### Parental Allocation

Prior to the parental allocation analysis, the power of the markers was assessed using 1,000 virtual F1 individuals. Using the 7 microsatellite set, a correct assignment of 92% was observed. Hence, we can consider the set of microsatellites to be informative enough for parental allocation procedures.

Parental allocations at 7 microsatellite loci for all the F1_V.I.P. individuals (N = 445) are summarized in Table C in File S1, including mitochondrial haplotypes used to solve ambiguities in case of multiple parental allocations. In total, 382 out of 445 individuals were successfully assigned to a single parent pair (30 families). No multi allocations were observed, which confirms the high resolution power of the markers. No discrepancies were observed between mitochondrial haplotypes and parental allocations. The remaining 63 individuals were not-allocated to any of the possible F0 parent pairs. However, four groups of full-sibs with different mitochondrial haplotypes were detected: Fam-unknownA (haplotype 3, 23 individuals), Fam-unknownB (haplotype 5, 4 individuals), Fam-unknownC (haplotype 2, 11 individuals) and Fam-unknownD (haplotype 6, 4 individuals). In total, the stock is composed of 30 families with known pedigree and at least 4 not-allocated families. Comparison of the F1 and F1-Alive stocks (Table C in File S1) showed the loss of few families, so that the F1-Alive stock is composed of 27 families of known pedigree and 3 not-allocated families (A and C were reduced to 11 and 5 individuals, respectively; B was unchanged; D was lost).

### Short-term breeding scheme (F0×F0)

**Figure 1:**
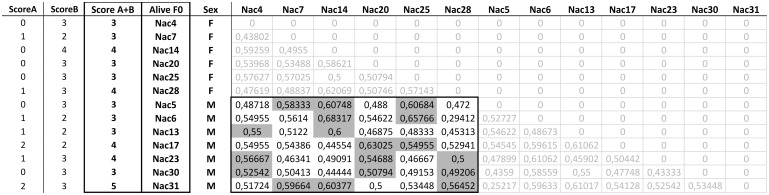
F0 breeding plan. Distance matrix at 24 microsatellite loci between the current F0 stock (Wild-Present) individuals. The already-existing families generated by each F0 parent and represented by at least 10 animals were counted (ScoreA). ScoreB represents the number of crosses per F0 that should be planned in order to get at least three families per breeder in the F1 generation (ScoreA+B). Only crosses between males and females are selected. Cells highlighted in grey indicate crosses selected for the short-term breeding plan on the basis of ScoreA and ScoreB.

A short-term breeding plan including the 13 F0 individuals still alive at present (7 males, 6 females) was designed after evaluation of the genetic relatedness of the individuals (Fig. 1). We aimed at having the contribution of each remaining F0 breeder in at least three families within the F1 generation. Mating pairs were selected among the more distant males and females based on the pairwise genetic distances at 24 loci (Fig. 1), trying to maximize the number of parents represented, with the minimum number of crosses. Following this strategy, 18 crosses were selected as priority (Fig. 1), among which only one was already present within the F1 stock with a limited number of individuals.

### Long-term breeding scheme (F1×F1) and selection of candidate breeders

**Figure 2:**
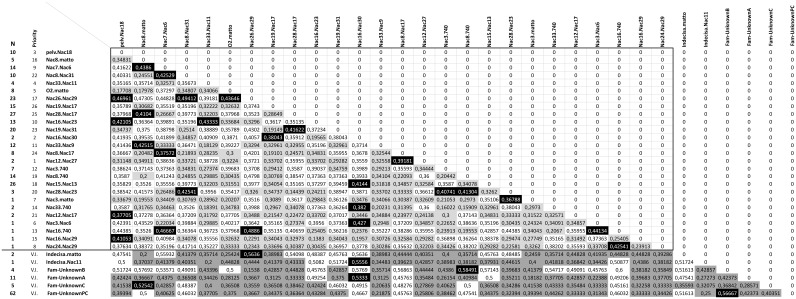
F1 breeding plan. Pairwise distances among the families of the “*Breeders Unit*” estimated by comparing the cumulative profiles of the parent pairs. The black frame includes distances estimated at 24 loci and excludes the ones based on 7 loci only. Light grey and dark grey cells represent crosses to be avoided based on threshold values of 0,35 (at 24 loci) and 0,45 (at 7 loci), respectively. Black cells represent selected crosses identified by the software “*BreedingPlanSturgeons*” (within the black frame) or by visual inspection (for the families marked with V.I.). N = number of individuals per family.

The long-term plan was designed based on the F1 progeny, using a family-based approach. A total of 32 crosses was planned, one for each of the 32 families that composed the “*breeders unit*”: 30 reared at V.I.P. (27 allocated and 3 not-allocated) plus two families from the Ticino River Park (1 allocated and 1 not-allocated). Pairwise genetic distances among families are shown in Fig. 2, together with details on recommended crosses and those crosses to be avoided (distance values <0.35 for 24 loci and <0.45 for 7 loci).

In the case of 4 not-allocated and 2 allocated families, generated by a single F0 female (reported in Fig. 2 as “Indecisa”) whose sample was accidentally loss, a reliable estimation of the genetic distances with the rest of families could not be achieved since they were only genotyped at 7 loci. Thus, these families were excluded from next simulations and only one cross was considered in order to guarantee their contribution to the next generation.

### Breeding plan simulations

**Figure 3:**
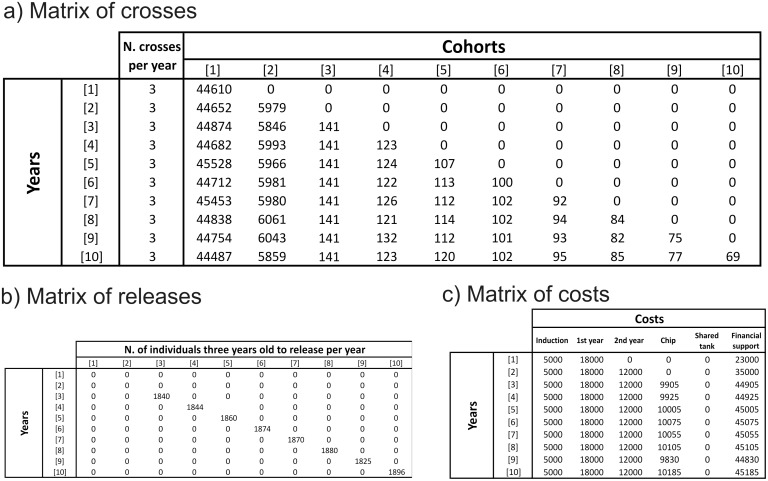
Example of output of the R-script “*CostsBreedingSturgeons*”. The run was performed using six tanks available for the first two years of rearing and one common pond of 1200 mq for the following years. (a) Number of individuals for each cohort (columns) maintained in captivity in the different years (lines). (b) Individuals that can be released per year. (c) Annual costs.

Results obtained from the simulations using the script “*CostsBreedingSturgeons*”, including inference of how many animals can be hosted in the facilities and released in the wild, as well as total costs of the project, are shown in Fig. 3.

Considering 6 ponds available at the facilities of Cassolnovo, where the progenies will be produced and reared, three crosses per year are feasible, which corresponds to about 45.000 larvae per year (15.000 per female). Induction of fish is not straightforward but assuming to induce 5 males and 5 females per cross, at least one successful reproduction is expected. Number of individuals at the 2^nd^ year dropped to ca. 6.000 individuals (Fig. 3a) after assuming a 86.7% mortality.

In order to save pedigree information, fingerlings produced by different family combinations should be kept separated for the first two years (until tagging). At the start of the 3^rd^ year, after tagging, the progeny is split into a fraction that is retained in a common tank with all other families to preserve the captive broodstock and a fraction that can be released into the wild. On the basis of a 10% yearly mortality, *ca.* 140 individuals are retained (Fig. 3a) in order to have an average of 20 individuals per family at the 10^th^ year of age. These individuals will constitute the “captive population” to be kept in the facilities as an “insurance policy” for the species. The remaining individuals (ca. 1.850) can be released into the wild (Fig. 3b). Finally, estimated costs are detailed in Fig. 3c, including costs generated by induction, maintenance and tagging of the individuals as well as total annual amount required for the breeding plan.

**Figure 4:**
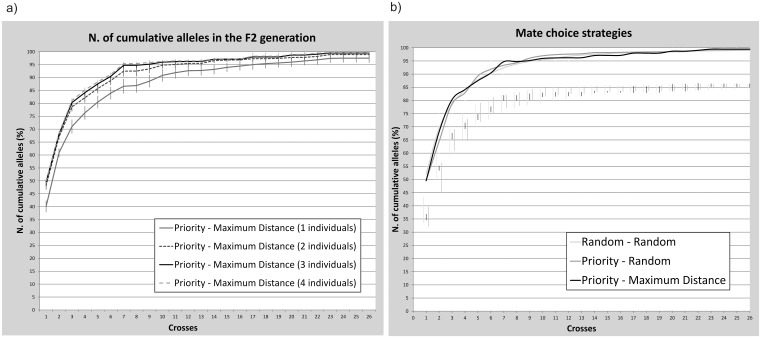
Simulations of allele inheritance under different strategies. Results of the simulations performed using the R-script “*BreedingPlanSturgeon*” to test: (a) the optimal number of breeders per family and (b) alternative strategies for the mating choice. The cumulative percentages of transmitted alleles are averaged on 100 replicates and reported with the corresponding standard deviation. In figure b, 3 individuals per family were crossed and standard deviation bars are shifted downwards for better visibility.

Results from simulations using the script “*BreedingPlanSturgeons*” to estimate the optimal number of breeders are presented in Fig. 4a, in which the cumulative number of alleles transmitted to the progeny is reported for different number of breeders. The fraction of alleles from the F0 generation that are successfully transmitted to the F2 is 100% when crossing either 2, 3 or 4 individuals per family. Only when crossing 1 single individual the transmission of alleles is not complete. The best strategy appears to be the use of 3 individuals, since the use of 2 individuals performed worst in the short-them, when the number of crosses was low.

Finally, results from simulations conducted to compare three alternative mating strategies are shown in Fig. 4b. While the rate of allele transmission was similar across mating strategies, both methods in which at least one family could be selected by chance (“Priority-Random” and “Random-Random”) showed larger standard deviations than the “Priority-Maximum Distance” method, particularly when the number of crosses was low, suggesting than the latter is the most conservative mating strategy.

## Discussion

We present the first breeding plan for the Adriatic sturgeon, a species which is believed to be possibly extinct in nature and whose future entirely relies on the implementation of an *ex situ* captive breeding plan based on genetic and demographic information upon which to establish management decisions. The program here proposed meets the two principals generally required in captive breeding: (i) the complete genetic characterization of all the individuals and (ii) the planning of a breeding scheme that minimizes genetic relatedness in order to maximize genetic variability. One particular characteristic of our breeding plan is that is “family-based” in contrast with most breeding plans that are “individual-based”. One of the difficulties when working with sturgeons is that adults are only reproductively active every other year or more rarely. This represents a serious problem since a selected individual might not be available for reproduction when needed. However, this problem can be avoided by using a family rather than a single individual approach, so that related individuals might be available instead.

### Characterization of the F1 V.I.P. stock

Pedigree reconstruction was applied to characterize the F1 stock retained at the V.I.P. plant and produced by crossing F0 individuals in the last 20 years. The approach implemented by the new software (“*BreedingSturgeons*”) and based on the inference of all possible genotypes compatible with observed band profiles, showed a higher allocation efficiency compared with the simple band sharing approach [10]. Accordingly, all ambiguities were completely solved with no F1 individual being multi-allocated. Comparison of genetic variability between the original F0 (Wild) and the F1 stock showed a relatively important loss of genetic diversity at the mitochondrial level, with 2 of the 7 original haplotypes being absent in the F1 stock before poisoning and a third one lost in the F1-alive group. However, at microsatellites, genetic diversity appears to be almost intact as no significant differences in number of alleles were observed between F0 and F1, with both samples presenting an average number of alleles per locus of about 10. By contrast, a lower value of 8.57 was reported for a different F1 stock of Adriatic sturgeon retained as possible future broodstock at the Ticino River Park [18]. The Ticino F1 stock was established without any genetic input and the animals were collected from a limited number of reproductions, causing a significant erosion of genetic diversity in a single generation, as exemplified by a 16.7% loss in average allele number in comparison with the F0 stock.

In synthesis, the V.I.P. Stock resulted to be the major source of variability for this species. Several families were identified and a great part of the F0 parents are represented within the stock. In regards to the relationships among animals, the probability to select for reproduction two related individuals by chance is very low (5,6%), compared to the 35% estimated for the stock reared by the Ticino River Park [18]. However, even if the use of Ticino F1 stock as *ex situ* broodstock is not advisable, this stock includes some families that are absent or under-represented in the V.I.P. plant, so exchange of individuals between plants should be encouraged.

Finally, our results also show how important is to test for the presence of hybrids or alien species. In many aquaculture facilities, different species or interspecific hybrids are reared, so that mixing of individuals can accidentally happen, and those can go unnoticed unless properly tested. In our case, two reciprocal hybrids between Adriatic and White sturgeon were found within the F1 progeny. As expected, hybrids showed microsatellite alleles not present in the *A. naccarii* F0 gene pool but these are not the only new alleles detected. Additional alleles not present in the parental generation were also found among not-allocated F1 individuals. The presence of new alleles in the F1 might be explained by inheritance from F0 breeders that died several years ago and are not present in our sample. Alternatively, F1 individuals carrying new alleles might be hybrids produced in the hatchery and accidentally mixed into the stock. Accordingly, accepting the risk of losing rare variants we chose to exclude from the broodstock all the animals carrying extra alleles.

### Is a short-term breeding plan still possible?

In the case of the Adriatic sturgeon, recruitment of new breeders from the wild is not possible since the species is possibly extinct in nature, so a breeding plan using wild breeders can only be achieved using the original F0 brought to the V.I.P. facilities in 1977. However, the F0 stock at the V.I.P. plant has been greatly reduced in size in recent years, probably due to senescence. While only 13 individuals remain at present, we propose a priority short-term breeding program to be attempted with these remaining F0 individuals. The plan includes 6 females and 7 males for a total of 18 selected crosses on the basis of pairwise genetic distance (Fig. 1). It should be noted that most of the remaining individuals had never been mated before, so that their successful reproduction would represent a relevant contribution to the future diversity of the captive stock of this species. Nevertheless, the fact that some animals have never been crossed in over 20 years of reproduction events might indicate a low reproductive potential. Hence, there is a chance that the proposed crosses between the surviving F0 adults might not be successful, and although we believe that a short-term plan using F0 should be attempted, most efforts should be conducted to the establishment of the long-term plan involving the F1 generation.

### A long-term breeding plan to safeguard the Adriatic sturgeon

We present a complete long-term breeding plan based on familiar groups that aims at generating enough animals to ultimately allow for the re-introduction of the species back into its natural environment. Considering the low chances of successful reproduction of these animals and their limited number, most efforts were devoted to developing a long-term breeding strategy involving families of F1 generation, assuming that at least some of the individuals are reproducible every year.

The program starts with the exclusion of combinations between families sharing one parent, together with families that do not share any parent but show low genetic distances, which might be indicative of relatives. This allows minimizing possible errors due to the “*founder assumption*”, in which wild founders of a captive population are assumed to be equally unrelated [30], although it might not always be the case.

Our choice of keeping separated for two years the animals produced by different family combinations, until the moment of tagging, limits the number of crosses that can be simultaneously performed with a finite number of ponds. However, it allows tracking the pedigree of every single animal providing useful information for monitoring the release program or for managing the following generation of captive broodstock. Alternatively, instead of crossing one individual per family, it could be considered to cross more individuals of the same family that can be pooled and do not require additional ponds. This would also increase the efficiency of allele transmission and guarantee that all F0 alleles of each family are inherited by the F2 generation, as shown by simulations that suggest the use of at least 3 breeders from the same family when possible. However, these numbers might be not easy to reach given the small size of some families and the discontinuous availability of breeders for reproduction (mostly true for females). Moreover, if breeders of the “*priority family*” are not available, one possibility is to postpone the cross until the following reproductive season and to slightly modify the priority order. If the unavailable individuals belong to the “*mating family*”, one possibility is to save the priority order and to select an alternative “*mating family*”, choosing the second more distant family. As shown by simulations, selection of family combinations based on priority and genetic distance were more efficient in transmitting the higher possible number of alleles to the next generation relative to the random strategies (“Priority – Random” and “Random – Random”). This was particularly true when number of crosses was low, which suggests that random approaches are not recommended for short-term programs. In fact, the higher standard deviation of the two strategies in which at least one family is randomly selected may result in a significant loss of allele diversity after few generation of captive breeding. Accordingly, the breeding plan here proposed was based on the Priority – Maximum distance approach. On one side, the establishment of a priority score allows to first cross those families whose genetic traits have the higher risk to be lost, since they are shared by a lower number of individuals in the population. On the other side, the use of maximum distance minimizes inbreeding and tends to increase the number of crosses involving families that are genetically peculiar, and consequently, are more often selected as “mating families”.

### Management implications

The present work represents the first breeding plan for the Adriatic sturgeon based on its detailed genetic characterization and can be considered as a reference guideline for all conservation actions based on controlled reproductions of this species. As in the case of Adriatic sturgeon, many other sturgeon species are critically endangered with greatly reduced wild populations and a consequent difficulty in collecting breeders from the wild. For such species the establishment of *ex situ* conservation plans through the retention of part of the fingerlings as future breeders is the only way forward. In this context, the approach here developed based on the management of familiar groups instead of individuals can become of major interest. Moreover, for those species that share with the Adriatic sturgeon the tetraploid condition, such as the Russian sturgeon (*A. gueldenstaedtii*), the Persian sturgeon (*A. persicus*), the Chinese sturgeon (*A. sinensis*) and many others, the methods here optimized are directly transferable. This would allow a careful identification of familiar groups thus providing the basis for the establishment of breeding programs that takes into account the genetic composition of the available broodstock together with logistic and economic constraints.

Our study also showcases the importance of genetic approaches to *ex situ* management for meeting conservation goals. The low cost of microsatellite markers facilitates the collection of genetic data that can inform breeding programs about the best pairing individuals so as to minimize mean relatedness. In our study, as few as 7 microsatellite markers were sufficient to unambiguously allocate all F1 individuals. In the event of higher number of putative parents, which might be the case in other sturgeon species, the number of microsatellites should be increased to ensure complete allocation.

Since genetic diversity is an essential prerequisite for the long-term adaptive potential, the availability of *ex situ* genetically heterogeneous broodstocks is the starting point for the rehabilitation of self-sustaining natural populations. However, in the short run, the survival rate of the released fingerlings depends on their capability do adapt to the target environment. In this sense, the establishment of adequate rearing protocols aimed to produce individuals with a high probability of survival is strongly encouraged. As clearly reported in FAO guidelines for sturgeon management and release [31], hatcheries involved in juvenile production for restocking purpose should raise fry and juveniles through training and adaptation to natural conditions, in order to maximize their fitness for survival after release.

As important as it is to use genetic studies into conservation practices, those need to be coordinated and not be carried out separately and restrictively. In the case of the Adriatic sturgeon, at present several local administrations are active in the attempt of recovering the species in its historical natural range. Unfortunately, these actions are often represented by short-term programs and are completely isolated and uncoordinated. Therefore, providing a common program like the one presented in our study that can connect and integrate the different actions would represent the first step towards a responsible concerted management of the Adriatic sturgeon. Finally, in order to implement successful conservation strategies, important actions against habitat degradation are urgently needed including the restoration of natural spawning sites and the re-establishment of river connectivity (i.e. fish passages at dams). A relevant challenge for conservation genetics is to go beyond the scientific since reintroduction is not merely a biological issue but a social, cultural and political one as well. An important part of the framework required for the successful incorporation of genetic data into conservation decision-making of culturally-important species is involvement where possible of local people in the program, together with public education about the project.

## Supporting Information

File S1 **Tables with additional details about the two R-scripts and samples analyzed are reported.** Tables A and B include a brief description of variables used in the two R-scripts. Table C reports details about results of parental allocations.
(DOCX)

File S2 **“** ***CostsBreedingSturgeons*** **” and “** ***BreedingPlanSturgeons*** **” scripts.** The zip file includes all system and executive files to run programs. As example, input files used to run “*BreedingPlanSturgeons*” script are also included.
(ZIP)

## References

[1] Leus K (2011) Captive breeding and conservation. Zool Middle East 54: 151–158.

[2] Witzenberger KA, Hochkirch A (2011) Ex situ conservation genetics: a review of molecular studies on the genetic consequences of captive breeding programs for endangered animal species. Biodivers Conserv 20: 1843–1861.

[3] Frankham R, Ballou JD, Briscoe DA (2002) Introduction to conservation genetics. Cambridge University Press, Cambridge, UK.

[4] Philippart JC (1995) Is captive breeding an effective solution for the preservation of endemic species? Biol Conserv 72: 281–295.

[5] Russello MA, Amato G (2004) Ex situ population management in the absence of pedigree information. Mol ecol 13: 2829–2840. 15315693 10.1111/j.1365-294X.2004.02266.x

[6] Araki H, Cooper B, Blouin MS (2007) Genetic effects of captive breeding cause a rapid, cumulative fitness decline in the wild. Science 318: 100–103. 17916734 10.1126/science.1145621

[7] Fraser DJ (2008) How well can captive breeding programs conserve biodiversity? A review of salmonids. Evol App 1: 535–586. 10.1111/j.1752-4571.2008.00036.x PMC3352391 25567798

[8] Kozfkay CC, Campbell MR, Heindel JA, Baker DJ, Kline P, et al (2008) A genetic evaluation of relatedness for broodstock management of captive, endangered Snake River sockeye salmon, *Oncorhynchus nerka* . Conserv Gens 9: 1421–1430.

[9] Goldstein PZ, DeSalle R, Amato G, Vogler AP (2000) Conservation genetics at the species boundary. Conserv Biol 14(1): 120–131.

[10] Congiu L, Pujolar JM, Forlani A, Cenadelli S, Dupanloup I, et al (2011) Managing polyploidy in ex situ conservation genetics: the case of the critically endangered Adriatic sturgeon (*Acipenser naccarii*). PLoS One 6: e18249. 21483472 10.1371/journal.pone.0018249 PMC3066226

[11] Jamie AI, Lacy RC (2012) A comparison of strategies for selecting breeding pairs to maximize genetic diversity retention in managed populations. J Hered 103: 186–196. 22246407 10.1093/jhered/esr129

[12] Hayes B, He J, Moen T, Bennewitz J (2006) Use of molecular markers to maximize diversity of founder populations for aquaculture breeding programs. Aquaculture 255: 573–578.

[13] Ludwig A (2006) A sturgeon view on conservation genetics. Eur J Wildlife Res 52: 3–8.

[14] Anders PJ, Drauch-Schreier A, Rodzen J, Powell MS, Narum S, et al (2011) A review of genetic evaluation tools for conservation and management of North American sturgeons: roles, benefits, and limitations. J App Ichthyol 27(Suppl (2)) 3–11.

[15] Wuertz S, Gaillard S, Barbisan F, Carle S, Congiu L, et al (2006) Extensive screening of sturgeon genomes by techniques revealed no sex-specific random screening marker. Aquaculture 258: 685–688.

[16] Fontana F, Lanfredi M, Chicca M, Congiu L, Tagliavini J, et al (1999) Fluorescent *in situ* hybridization with rDNA probes on chromosomes of *Acipenser ruthenus* and *Acipenser naccarii* (Osteichthyes Acipenseriformes). Genome 42: 1008–1012.

[17] Bronzi P, Rosenthal H, Arlati G, Williot P (1999) A brief overview on the status and prospects of sturgeon farming in Western and Central Europe. J App Ichthyol 15: 224–227.

[18] Boscari E, Congiu L (2014) The need for genetic support in restocking activities and *ex situ* conservation programmes: the case of the Adriatic sturgeon (*Acipenser naccarii* Bonaparte, 1836) in the Ticino River Park. J App Ichthyol DOI:10.1111/jai.12545.

[19] Boscari E, Barmintseva A, Pujolar JM, Doukakis P, Mugue N, et al.. (2014) Species and hybrid identification of sturgeon caviar: a new molecular approach to detect illegal trade. Mol Ecol Res doi:10.1111/1755-0998.12203. 10.1111/1755-0998.12203 24219811

[20] Tamura K, Peterson D, Peterson N, Stecher G, Nei M, et al (2011) MEGA5: Molecular Evolutionary Genetics Analysis using Maximum Likelihood, Evolutionary Distance, and Maximum Parsimony Methods. Mol Biol Evol 28: 2731–2739. 21546353 10.1093/molbev/msr121 PMC3203626

[21] Clement M, Posada D, Candrall KA (2000) TCS: a computer program to estimate gene genealogies. Mol Ecol 9: 1657–1659. 11050560 10.1046/j.1365-294x.2000.01020.x

[22] Templeton AR, Crandall KA, Sing CF (1992) A cladistics analysis of phenotypic associations with haplotypes inferred from restriction endonuclease mapping and DNA sequence data. III. Cladogram estimation. Genetics 132: 619–633. 1385266 10.1093/genetics/132.2.619 PMC1205162

[23] Librado NP, Rozas J (2009) DnaSP v5: a software for comprehensive analysis of DNA polymorphism data. Bioinformatics 25: 1451–1452. 19346325 10.1093/bioinformatics/btp187

[24] May B, Krueger CC, Kincaid HL (1997) Genetic variation at microsatellite loci in sturgeon: primer sequence homology in *Acipenser* and *Scaphirynchus* . Canadian Journal of Fisheries and Aquatic Science 5: 1542–1547.

[25] Forlani A, Fontana F, Congiu L (2007) Isolation of microsatellite loci from the endemic and endangered sturgeon *Acipenser naccarii* . Conser Gen 9: 461–463.

[26] Zane L, Patarnello T, Ludwig A, Fontana F, Congiu L (2002) Isolation and characterization of microsatellite in the Adriatic sturgeon (*Acipenser naccarii*). Mol Ecol Notes 2: 586–589.

[27] Henderson-Arzapalo A, King TL (2002) Novel microsatellite markers for Atlantic sturgeon (*Acipenser oxyrinchus*) population delineation and broodstock management. Mol Ecol Notes 2: 437–439.

[28] Boscari E, Barbisan F, Congiu L (2011) Inheritance pattern of microsatellite loci in the polyploidy Adriatic sturgeon (*Acipenser naccarii*). Aquaculture 321: 223–229.

[29] Hood GM (2010) PopTools version 3.2.5. Available: http://www.poptools.org. Accessed 2014 October 10.

[30] Van Dyke F (2008) Conservation biology: foundations, concepts, applications. Wheaton College, Illinois, USA. Second Editions, Springer Science and Business Media.

[31] Chebanov M, Rosenthal H, Gessner J, Van Anrooy R, Doukakis P, et al (2011) Sturgeon hatchery practices and management for release-Guidelines FAO Fisheries and Aquaculture Technical Paper No 570. Ankara, FAO. 2011: 110.

