# Supplementary material for: Captive Breeding Programs Based on Family Groups in Polyploid Sturgeons
Source: PLoS One. 2014 Oct 30;9(10):e110951. doi: 10.1371/journal.pone.0110951 (PMC4214717; doi:10.1371/journal.pone.0110951)
Supplement: File S1 — Tables with additional details about the two R-scripts and samples analyzed are reported. Tables A and B include a brief description of variables used in the two R-scripts. Table C reports details about results of parental allocations. (DOCX) [file pone.0110951.s001.docx]

**Supplementary materials**

| **Script “CostsBreedingSturgeons”** | | |
| --- | --- | --- |
| **Variable** | **Description** | **Values** |
| **nTank** | Number of ponds available for rearing the families separately until tagging | 6 |
| **nInduction** | Number of individuals that can be induced every year per sex | 5 |
| **nyears** | Total number of years considered for the breeding plan | 10 |
| **nFinal** | Final number of adults to be retained as future breeders per family | 20 |
| **maleProb** | Frequency of successful induction in males | 0.7 |
| **femaleProb** | Frequency of successful induction in females | 0.6 |
| **firstYearSurvProb** | Survival rate in the first year | 0.133333 |
| **secondYearSurvProb** | Survival rate in the second year | 0.333333 |
| **YearSurvProb** | Annual survival rate after the second year | 0.9 |
| **nEggs** | Average number of eggs per induced female | 30.000 |
| **openProb** | Hatching rate | 0.5 |
| **maxBiomax** | Maximum available space in the common pond (m2) | 1200000 |
| **inducCost** | Estimated cost for the induction of 10 individuals | 5.000 |
| **firstYearCost** | Management costs* for the first year per family (euros) | 6.000 |
| **secondYearCost** | Management costs* for the second year per family (euros) | 4.000 |
| **chipCost** | Tagging costs per individual | 5 |
| **finalTankCost** | Costs of final tank management | - |

**Table A. Variables of the R-script “*CostsBreedingSturgeons*”.**

Used values, referred to the “Storioni Ticino” hatchery, are reported in the last column.

| **Script “BreedingPlanSturgeons”** | |
| --- | --- |
| **Variable** | **Description** |
| **nFam** | Number of families included in the breeders unit |
| **nInc** | Number of crosses to perform per family |
| **famMethod** | Method for the selection of the “priority family”. **“Prior” or “Random”** |
| **sampling** | Method for the selection of the “mating family”. **“distance” or “random”** |
| **nRep** | Number of replicates |
| **nIndprior** | number of individuals to cross from the “priority family” |
| **nIndinc** | number of individuals to cross from the “mating family” |
| **nSons** | number of virtual offspring to generate per family |
| **limit** | In the case of selection of “mating family” by “distance”, maximum number of crosses in which a mating family can be used. |

**Table B.** **Variables of the R-script “*BreedingPlanSturgeons*”.**

Description of variables used in the R-script “*BreedingPlanSturgeons*”.

| Stock | | | | |
| --- | --- | --- | --- | --- |
| F1 | **F1-Alive** | **F1 individuals** | **Parental pairs** | **Haplotypes** |
| 11 | 2 | N2882B, N0645, N1245, N2153, **N3432**, N4418, N6091, N7251, N9027, N9181, **N9860** | **Pelvienne (F) x NaccS18 (M)** | 2 |
| 9 | 5 | **N0187, N0911, N1671**, N5520, N6045, **N7547**, N7654, N7688, **N8595** | **NaccS8 (F) x Matto (M)** | 2 |
| 14 | 14 | **N0983, N1349, N3180, N4021, N4364, N6373, N6670, N6868, N7326, N7755, N7808, N8390, N9726, N9729** | **NaccS7 (F) x NaccS6 (M)** | 3 |
| 30 | 10 | N0964, N1939, N2738, N4150, N4453, N4868, **N7413, N7897**, N8029, N8101, N8361, N9493, N2490, **N5779, N0952, N1734, N6057,** N6931, **N7911**, N0303, **N0749**, N1094, **N2274**, N4263, N5268, N5468, N6096, N6842, **N9295**, N5734 | **NaccS8 (F) x NaccS31 (M)** | 2 |
| 10 | 4 | N9837, N3918, **N4322, N6584, N7772, N9926,** N1400, N4143, N4395, N4504 | **NaccS33 (F) x NaccS11 (M)** | 3 |
| 12 | 8 | N1167, N1174, **N1401**, N1515, **N2235, N2260**, N4109, **N5453, N5475, N6197, N8290, N9563** | **O2 (F) x Matto (M)** | 5 |
| 32 | 23 | **N0675, N1790, N5660**, N6727, **N1540, N2142**, N2718, **N5209**, N5739, **N0237, N1221, N1255**, N1267, **N1533**, **N2268, N3009, N3201**, N4821, **N5212, N5280**, N5630, **N5680, N5728, N5904**, N6219, **N6579, N6784**, N8147, **N8850, N9510, N9953**, N8234 | **NaccS26 (F) x NaccS29 (M)** | 5 |
| 22 | 15 | N1780, **N1921, N4450, N6500, N9009, N9955,** N0268, **N1713, N3764, N3928, N4652**, N4850, **N4940, N5150**, N5175, **N5474, N5566**, N6059, N7549, **N7909, N9256**, N0786 | **NaccS19 (F) x NaccS17 (M)** | 5/7 |
| 48 | 27 | **N0203, N0291, N0319, N0550,** N0691, **N1077,** N1426, **N1478**, N1561, N1768, **N2122, N2148**, N2538, N3055, N3920, N3924, **N4157, N4313, N4801**, N5919, **N5941, N5992, N6006, N7510, N7611,** N7670, **N7753, N8091, N8199**, N8688, N8836, **N9074**, N9521, **N23000**, N7781, **N0890, N1289, N4167**, N6024, **N0968, N1911, N2082**, N7895, N9066, N2573, N8771, N6181, N1764 | **NaccS28 (F) x NaccS17 (M)** | 3 |
| 38 | 13 | N0371, **N1772**, N2037, **N2566, N2703**, N2780, N2999, N3226, N3729, **N3861**, N4139, N4975**, N5208**, N5382, N5554, N5854, N6343, **N6703, N6765**, N7264, N7336, **N7383**, N7554, **N7629,** N7726, **N8338**, N8713**, N8777**, N8797, N9298**, N9346**, N9613, N2129, **N3446**, N8939B, N3947, N7738, N8318 | **NaccS16 (F) x NaccS23 (M)** | 3 |
| 31 | 20 | **N0148, N1827, N7230**, N7373, N7530A, **N8189**, N0046, **N0679**, N1157, N1386**, N1390, N2697**, N2742, **N3101**, N3458, **N4991, N5470, N5486, N6092, N6516**, N6753, N7099, **N7423, N7509**, N8158**, N8892, N9025**, N9230, **N9303, N9569, N9987** | **NaccS19 (F) x NaccS31 (M)** | 5/7 |
| 13 | 2 | N0965, N3813, N8203, N8815, **N9134**, N0808, **N3525**, N4356, N4398, N4670, N4920, N6509, N8312 | **NaccS16 (F) x NaccS30 (M)** | 3 |
| 17 | 12 | **N2254**, N2955, **N5254**, N7250, N8549, **N9103, N0699, N1511, M2242, N2256, N2843, N5826, N5884, N6200, N7107**, N8129, N8854 | **NaccS33 (F) x NaccS9 (M)** | 3 |
| 15 | 8 | **N1923, N4544**, N5945, **N6741**, N9962, **N0265**, N1563, **N3758, N4100**, N4214, **N5571**, N5743, **N6069**, N7798, N0807 | **NaccS8 (F) x NaccS17 (M)** | 2 |
| 10 | 2 | N0461, N0473, N1131, N7374, N8939A, **N6644**, N7474, N2059, **N3264**, N6962 | **NaccS12 (F) x NaccS27 (M)** | 2 |
| 24 | 7 | **N4503, N4591**, N7530B, N7577, **N8818**, N8507, **N0841, N1357**, N1984, N2542, **N3522**, N3596, **N3648,** N4145, N6064, N6249, N6414, N6592, N7114, N7556, N8146, N8187, N8426, N8576 | **NaccS3 (F) x 740 (M)** | 3 |
| 21 | 14 | **N6730, N0625, N4411, N6511, N6884**, N9179, **N1934, N2757**, N0393, N4023, **N4282, N5183**, N5967, N6041, **N7112**, N7706, **N9048, N9255**, N9480, **N9750, N0934** | **NaccS8 (F) x 740 (M)** | 2 |
| 3 | 3 | **N2689, N4883, N9367** | **NaccS28 (F) x NaccS23 (M)** | 3 |
| 4 | 1 | N4650, **N5733**, N6550, N7113 | **NaccS3 (F) x Matto (M)** | 3 |
| 5 | 2 | **N8113**, N0280, N3589, **N3668**, N9371 | **NaccS33 (F) x 740 (M)** | 3 |
| 2 | - | N0648, N0549 | **NaccS7 (F) x VerdeBis (M)** | 3 |
| 2 | 1 | **N9786**, N0049 | **NaccS12 (F) x NaccS17 (M)** | 2 |
| 2 | 2 | **N4295, N4443** | **Indecisa (F) x Matto (M)** | 2 |
| 1 | 1 | **N5519** | **Indecisa (F) x NaccS11 (M)** | 2 |
| 1 | - | N8097 | **NaccS12 (F) x Matto (M)** | 2 |
| 1 | 1 | **N6713** | **NaccS3 (F) x NaccS6 (M)** | 3 |
| 1 | - | N0542 | **NaccS3 (F) x NaccS29 (M)** | 3 |
| 1 | 1 | **N9523** | **NaccS16 (F) x 740 (M)** | 3 |
| 1 | 1 | **N3383** | **NaccS16 (F) x NaccS29 (M)** | 3 |
| 1 | 1 | **N3447** | **NaccS24 (F) x NaccS29 (M)** | 5 |
| 63 | 33 | **N0369, N4038, N4743, N4964**, N7358, **N7715, N7730**, N9298B, N9590, N1055, **N1163**, N1716, N2882A, **N3547**, N3843, N4123, N4155, **N4511**, N4955, **N5386, N5407, N5469**, N6186, N7101, **N7543**, N7641, N9227, N0160, N0463, N0673, N0702, N0855, **N0857**, N1345, **N1673**, **N1997**, N2165, N2197, N2824, **N2854**, N3568, **N3655, N3847, N4125**, N4305, N4454**, N5981, N6242**, **N6769, N7084**, N7652, N7886, N7921, **N8201, N8793**, **N9056**, N9373, **N9507, N9824, N2062, N5914, N8123, N8991** | **Not allocated** | 2-3-5-6 |

**Table C.** **Parental allocation analysis.**

Results of parental allocation of F1 individuals to the F0 generation. Number of F1 and F1-alive individuals and mitochondrial haplotype are also reported. Each individual is identified by microchip code (individuals alive at present are in bold). Among non-allocated individuals, detected hybrids are underlined.
